# Reconciling security and care in digital medicine

**DOI:** 10.1038/s41746-025-01685-0

**Published:** 2025-05-08

**Authors:** Chiara Carboni, Celia Brightwell, Orit Halpern, Oscar Freyer, Stephen Gilbert

**Affiliations:** 1 Chair of Digital Cultures, School of Humanities and Social Sciences, TU Dresden, Dresden, Saxony Germany; 2 Else Kröner Fresenius Center for Digital Health, Medical Faculty, TU Dresden, Dresden, Saxony Germany

**Keywords:** Health care, Health policy, Health services, Public health, Sociology

## Abstract

Common approaches to cybersecurity frame end users as the weakest link in a system. In this Perspective, we argue that appraising end users as contributors to security can help reconcile both security and care practices. Through two case studies, including Swedish hospitals and a UK smart-home system, we demonstrate the gaps between established security protocols and the daily reality of care practices, and outline approaches to reconcile security and care.

## Security paradigms in digital medicine

The digitization of healthcare introduces new vulnerabilities such as the risk of data breaches and cyber attacks^1^, which have serious consequences for healthcare infrastructure and patient safety and privacy^2,3^. The response has often been to maximize security from a technical standpoint^4^, focusing on the modalities of data collection, the robustness of systems to downtime, and approaches to ensure cybersecurity. Recent information security paradigms such as security-by-design aim at “building security into” digital health technologies^5^. In practice, this can mean security measures such as strict user authentication are difficult and time-consuming for end users, making security a barrier to healthcare efficiency and quality^5^. This Perspective calls attention to how an emphasis on technical security risks, without concern for socio-medical practices, introduces a chasm between care practices and the digital technologies used to support these practices. We argue that there is a need to reconcile the technical and social elements of security and care. For instance, getting healthcare systems to perform as needed often requires end users to circumnavigate security measures such as authentication^6^. This leads to a paradoxical situation whereby concerns and practices aimed at achieving effective patient care are in turn considered as barriers to security-compliant behavior^7^.

When end users are factored into discussions of digital health security, they are often framed as vulnerabilities^8^ or the weakest link^9^ in the system. This reflects cybersecurity discourse in general, which frames individual human actors as a “problem” in the technical system^10^. For example, end users are framed as systemic weaknesses that might expose networks to malicious attacks^11^, and their interactions with systems are framed as antagonistic to infrastructure and data security^12,13^. This framing of end users leads to information-security approaches based on control-based compliance models, which seek to regulate human behavior through bureaucratic security rules^10,14^. Failing to acknowledge that non-compliance does not necessarily stem from a lack of knowledge, and instead may be the result of informed decisions that privilege different rationalities, values, and aims^15^ can lead to less effective solutions. For instance, further security training is often prescribed for healthcare workforce training^9^ instead of exploring the root causes of non-compliance. Among other factors, this approach – in which end users are framed as a problem, and security processes are designed to minimize their interaction – “does not seem to work”^10^ given the ongoing escalation of cyber attacks, of which digital healthcare infrastructure is a prime target^16^.

While we question the effectiveness of framing end users as vulnerabilities, we do not deny that large numbers of data and cybersecurity breaches can be traced back to end users. However, the increasing emphasis on technical security in digital care systems urges a reflection on what is to become of care values that are cast as antithetical to secure systems. Indeed, research in the social sciences has previously argued for the irreconcilability of these two logics. The UK’s National Health Service (NHS) Federated Data Platform, developed on Palantir’s data analysis platform Foundry, has recently been criticized for importing into the healthcare domain values that are extrinsic to care, such as technical efficiency^17^. Philosopher Marjolein Lanzing highlights the risk that the “sphere of health will increasingly be structured according to the industrial logic and values of the sphere of security, crowding out those of care, changing its meaning”^17^. This highlights the reality that technologies are not neutral, and often encode social, political, and economic values.

Responding to this situation, this Perspective explores how, and under which conditions, security might be reconciled with, and indeed made an integral part of, care in digital health. It proposes that a way forward might lie in building security frameworks and practices that are shaped by care, rather than in opposition to it. As political scientists Berenice Fisher and Joan Tronto suggest, care includes “everything that we do to maintain, continue and repair ‘our world’ so that we can live in it as well as possible”^18^. This concept of care refers to practices that are “ambivalent, contextual, and relational”^19^, thus emphasizing that maintaining the world is a matter of fostering relationships and navigating clashing commitments (i.e., different ideas about what is good and necessary), rather than formulating rules and principles^20,21^. Scholarship in the social sciences has also argued that technologies should not be conceived of as opposed to care, but rather an integral part of it^22,23^.

This Perspective transposes this argument on the relationship between care and technology to the context of care and security. It argues that reconciling security and care in digital health entails at least three connected steps. First, looking at care practices as sources of inspiration for innovating security approaches and frameworks. Second, appraising end users as potential contributors to the security of connected medical devices. Third, engaging end users in security processes for digital health through participatory design.

Methodologically, this Perspective substantiates these points by drawing on two qualitative case studies. These case studies were selected from secondary literature due to their ability to capture the tensions and overlap between logics of security and logics of care. They foreground, respectively, the organizational and professional perspective, and the perspective of older patients, often framed as a vulnerability in security discourse due to their (assumed) limited digital literacy. Although we provide more context for each case below, both cases mobilize observational qualitative research designs. The first one aims at understanding “the rationalities drawn upon by healthcare professionals in their information security practice”^14^, hinging on a comparison between information security actions (ISAs) as they are prescribed in information security documents and as they are implemented by professionals in their daily work. The authors map prescribed and actual ISAs, respectively, by analyzing information security policy documents and by observing professionals in their daily work, and especially in their interactions with electronic health records and electronic communication platforms. Interviews with high-level information security managers (*n* = 3) and with clinicians (*n* = 24) were used to illuminate the value-based rationales behind, respectively, prescribed and actual ISAs, especially when the two diverged. The second case harnesses four focus groups with older adults (*n* = 13) who had been involved in the 12-month pilot of a smart-home system. The authors sought to investigate “older adults’ perceptions and expectations of smart home technology as well as their beliefs about the ways in which technology could help to improve their daily lives”^24^. Holding the focus groups in a lab where the system’s prototype had been installed enabled the researchers to both “foster participants’ confidence in their ability to be creative and useful when talking about technology” and to focus the discussion “on real-world examples”^24^.

### Case study: Information security in Swedish hospitals

The first case study focuses on the gaps between security protocols and care practices in healthcare organizations^14^. Access to digital healthcare systems such as medical records is governed through a range of information security measures, such as password creation and protection guidelines, and authorized access protocols. Hedström et al. illustrate how everyday care practices in two small Swedish county hospitals diverge from prescribed security practices.

For example, information security protocols in the hospital state that passwords should not be shared. In reality, to make it easier to access passwords required for different electronic systems, caregivers wrote passwords on pieces of paper they stuck above their computers. In another case, the password to access an electronic system was written on a consultation room’s wall. Similarly, an information security protocol stated that only authorized people should be able to access a system. In reality, the system for patient registration was kept logged on and accessed by all members of staff, rather than each staff member logging off and re-authenticating each time. In these examples, healthcare professionals’ easy access to necessary patient information led to them circumnavigating the information security protocols.

In contrast, an information integrity and availability protocol states that patient information must be documented at once when a caregiver is with the patient. In reality, a physician waits to document the patient’s information because he decides it is not needed immediately. A similar assessment is made by a counselor, who sometimes decides not to enter particularly sensitive information in a patient’s medical record due to respect for the patient’s privacy. The counselor explains that the patients communicate sensitive information to her in a trusted situation, while the medical records can be read by other groups of users who do not necessarily have the same trusting relationship with the patient as the counselor.

The researchers observed how healthcare staff made informed decisions that negotiated both security and care practices in a way that required circumnavigating the information security and integrity protocols:

“More sensitive information (i.e., patient information in the medical records) was well protected by the actions of the health care staff while less sensitive information (i.e., lists showing patients coming to the clinic that day) was made available to all”^14^.

The hospital staff members demonstrate a nuanced and contextual understanding of what constitutes ‘sensitive’ data – omitting very private information from the medical record, while making other information easily available – that contrasts with the documented security protocols. Hedström et al.’s observations show that there is no way to take into account actual care practices without changing ideas of what information can be securely shared, since there is no such thing as care without information sharing. Therefore, acknowledging the expertise and sensitivity hospital staff demonstrate in their information security practices is critical to improving security protocols. The authors suggest protocols should be designed and implemented with hospital staff, rather than a top-down process in which security teams create protocols that care teams are expected to comply with.

### Case study: Security and care in the SPHERE smart home

The second case study illustrates how patients’ experiences of care and security can come into conflict. The SPHERE (a Sensor Platform for Healthcare in a Residential Environment) smart-home system comprises home-based sensors designed to detect changes in health or well-being in older people living at home^24^. It includes sensors that monitor humidity, temperature, air quality, noise, light, occupancy, door contacts, and water and electricity consumption^24^. In Ghorayeb et al.’s qualitative study of older adults’ perspectives on the technology, most participants appear to have an overall positive relationship to the SPHERE system. However, the researchers surfaced an awareness of cybersecurity risks that threaten to intervene in the system’s care function. An interview with one potential user revealed the concern that the SPHERE system could make them a target for hackers:

“It’s all intelligent data which they collect, and all this data it is so easy to identify the vulnerable people and I think that’s where my problems would be… I mean if it got in the wrong hands then what would happen is [a] lot of people would realize that this person is vulnerable and lives on his own and you know what happens with vulnerable people!”^24^.

This potential user’s repeated description of themselves as ‘vulnerable’ highlights the complex relationship between security and care in the smart home. Even in the absence of a cyberattack, the system introduced other forms of insecurity that might shape users’ engagement with the technology. Participants described receiving marketing materials for holiday brochures and funeral plans that they believed targeted them based on their age, and expressed concern about how the SPHERE system may further mark their vulnerability to malicious actors. This reveals a tradeoff between the extra sense of care participants can feel due to the SPHERE system, and the new sense of insecurity they feel based on fears regarding data privacy breaches and sale of patient data to third parties. Although the researchers register users’ concerns, they do not explore how they might be reflected in how users use the system. As we discuss below, harnessing the security practices users develop in interaction with systems they perceive as insecure might provide a path towards reconciling security and care logics.

### End users as solution in secure care systems

This Perspective has built on recent calls for human-centric approaches to cybersecurity in the field of computer science^25,26^, as well as on studies that have pointed out the tensions between values driving the logic of security and the logic of care^14,15^. As we have argued, reconciling security with care requires three connected steps.

First, looking at care practices as sources of inspiration for innovating security approaches and frameworks. In this sense, the concept of exnovation might be useful. Exnovation proposes an alternative approach to innovation that, rather than simply replacing existing practices, focuses on “the hidden competences of practices and practitioners”^27^. Importantly, the related realm of patient safety has recently advocated and largely implemented a similar transition, from so-called Safety-I to Safety-II approaches. Whereas Safety-I treats humans as liabilities and emphasizes the importance of strict compliance with safety protocols, Safety-II frameworks emphasize the complexity of healthcare environments, and how humans’ ability to “adjust what they do to match the conditions of work” represents an asset, rather than a hazard^28^. Likewise, in a security context, an exnovation approach suggests focusing not so much on what goes wrong in practice, but on what people do manage to achieve even in difficult or insecure situations^27^. This involves acknowledging that security practices, as well as care practices, are “ambivalent, contextual, and relational”^19^. Concretely, this requires ongoing attention to digital health technology use within everyday care practices to understand how inherent competences developed by users in practice might be harnessed to achieve security frameworks that are more compatible with care. For example, despite circumnavigating information security protocols, the Swedish hospital staff in the first case study nevertheless developed security practices that were both compatible with their care practices and protected what they considered to be sensitive information. As a model that facilitates reconciling care and security practices, Hedström et al suggest moving away from the current focus on security compliance materializing in “sanctions, controls and regulation of users”^14^. Rather, they suggest acknowledging that “behavioral information security problems” often stem from a conflict in the values informing, respectively, mandated security protocols and care practices. Acknowledging these value tensions might open up avenues to put care and security into conversation.

Second, and related, reconciling security and care involves appraising end users, such as healthcare professionals and patients as shown in the two case studies, as potential contributors to the security of connected medical devices. For example, a potential failure to reconcile the technical and social aspects of security in the SPHERE smart home showed how a technical system intended to enhance the social sense of security risked doing the opposite. The participant felt the system marked them as vulnerable, and that a data privacy breach could make them or their home a target for burglars. Re-framing the effectiveness of digital health technologies as always entangled with the input of patients, human caregivers, and other actors is necessary to understand how this technology affects care experiences, and for policy that anticipates security concerns in connected healthcare systems. By treating end users as contributors to system security, these technologies can more effectively harness human behavior to enhance both security and care outcomes.

Third, engaging end users to help shape security processes from the beginning is possible through participatory design approaches. This could help capture the “invisible work”^29^ required of end users and their contributions to making these systems effective and secure. Sociological perspectives use the concept of end users’ invisible work to show how digital health technologies redistribute, rather than reduce, work^29^ and ultimately transform care practices^30^. Participatory design approaches that collaborate with end users could identify value conflicts and points of tension between care and security mandates to produce security processes that work well in everyday care practices, not just in theory. Notably, participatory design diverges from approaches such as “usable security”^31^ or the FDA-prescribed usability testing^32^ in that it casts a deeper and more constructive focus on user practices, goals, and needs, rather than zeroing in on errors as threats to the integrity and effectiveness of the system. Indeed, participatory design has often been advocated to improve the usefulness, ease of use and acceptability of digital health interventions^33,34^, as well as to navigate conflicting needs of different stakeholders^35^. Participatory design is predicated on reaching a deep understanding of end users’ goals, values and needs through iterative approaches that involve them “as expert on their own experience” in an ongoing manner^36^. Not incidentally, the FDA has recently acknowledged the need for similar approaches in the launch of their Health Care at Home Initiative^37^. Although we are not aware of examples in the digital medicine realm, researchers have identified the potential for participatory design approaches to cybersecurity processes for non-healthcare contexts. For example, this approach has helped challenge “top-down and siloed thinking” from security experts in the UK’s Digital Security by Design program^38^, and highlighted alternatives to Western-centric cybersecurity paradigms in rural Africa^39^. We identify an opportunity for future research to explore a participatory design approach to cybersecurity processes in digital health contexts. More broadly, future research on the entanglement of security and care practices in digital health can address how and why these systems are digitized, how these systems are impacted by the scale and economy of data, which incentives and disincentives exist to stop cyber criminals, and how security can be understood in terms of care and health outcomes.

Underpinning these three steps is a recognition of the ultimate impossibility of building perfectly secure systems for highly volatile, messy and unpredictable environments such as healthcare settings^40^. Resilient healthcare systems must empower end users to anticipate, detect, and respond to potential security incidents^41^. By keeping humans ‘in the loop’^10^ and enabling them to adapt to emerging situations, digital health systems can better balance both care and security, allowing for more effective responses to anomalies and greater system stability.

Given the decades-long systematic dismantling of public health services in Europe^42,43^ and OECD countries in general^44^, this Perspective’s articulated strategies for reconciling security and care are likely to encounter practical hindrances. Admittedly, constrained budgets, staff shortages and other socio-economic tensions currently exerting pressure on care provision might hamper the implementation of time- and resource-intensive interventions such as participatory design. Nonetheless, it is crucial that the current emphasis on securing digital health does not translate into a further dismantling of care infrastructures and practices. To this end, this Perspective has sought to emphasize the necessity of a reappraisal of attitudes and assumptions concerning end users in security frameworks, practices, and protocols in healthcare. Ultimately, as we have argued, it behooves us to start shifting the conversation on security and digital health, to fully appreciate and work with the tensions in values and the risk allocation negotiations that shape end users’ practices and experiences of care and security. Ultimately, there can be no security without care.

## Data Availability

No datasets were generated or analysed during the current study.

## References

[1] Tully, H., Coravos, A., Doerr, M. & Dameff, C. Connected medical technology and cybersecurity informed consent: a new paradigm. *J. Med. Internet Res.* **22**, 3 (2020). 10.2196/17612 PMC7154933 32224492

[2] Eddy, M. & Perlroth, N. Cyber attack suspected in German woman’s death. *New York Times*. www.nytimes.com/2020/09/18/world/europe/cyber-attack-germany-ransomeware-death.html (2020).

[3] Gilbert, S. et al. Can we learn from an imagined ransomware attack on a hospital at home platform?. *npj Digital Med.* **7**, 65 (2024). 10.1038/s41746-024-01044-5 PMC10954698 38509171

[4] Li, Z., Nam, T. hanh, Vu, S., Dragoni, N. & Doherty, K. Accomplishing more with less: the practice of cybersecure health technology design among Danish startups. *CHI: Conf. Hum. Factors Comput. Syst.* **21**, 1–8 (2023).

[5] Bernsmed, K. & Jaatun, M. G. Security-by-design challenges for medical device manufacturers. *Eur. Interdiscip. Cybersecurity Conf.* 155–160 (2024).

[6] Stobert, E., Barrera, D., Homier, V., Kollek, D. Understanding cybersecurity practices in emergency departments. *CHI: Conf. Human Factors Comput. Syst.* 1–8. (2020).

[7] Coventry, L. et al. Cyber-risk in healthcare: exploring facilitators and barriers to secure behaviour. In *HCI for Cybersecurity, Privacy and* Trust (ed Moallem, A) 105–122 (Springer, 2020).

[8] Pollini et al. Leveraging human factors in cybersecurity: an integrated methodological approach. *Cognit. Technol. Work* **24**, 371–390 (2022). 34149309 10.1007/s10111-021-00683-y PMC8195225

[9] Fotis, T., Kioskli, K. & Mouratidis, H. Human factors and cybersecurity in NHS Virtual Wards. *Hum. Factors Cybersecur.* **127**, 225–235 (2024).

[10] Zimmermann, V. & Renaud, K. Moving from a ‘human-as-problem’ to a ‘human-as-solution’ cybersecurity mindset. *Int. J. Hum. Comput. Stud.* **131**, 169–187 (2019).

[11] Klimburg-Witjes, N. & Wentland, A. Hacking humans? Social engineering and the construction of the “deficient user” in cybersecurity discourses. *Sci. Technol. Hum. Values* **46**, 1316–1339 (2021).

[12] Koppel, R., Smith, S., Blythe, J. & Kothari, V. Workarounds to computer access in healthcare organizations: you want my password or a dead patient?. *Stud. Health Technol. Inform.* **208**, 215–220 (2015). 25676976

[13] Marchang, J. et al. Secure-by-design real-time internet of medical things architecture: e-Health population monitoring. *Telecom* **5**, 609–631 (2024).

[14] Hedström, K., Kolkowska, E., Karlsson, F. & Allen, J. P. Value conflicts for information security management. *J. Strateg. Inf. Syst.* **20**, 373–384 (2011).

[15] Loi, M., Christen, M., Kleine, N. & Weber, K. Cybersecurity in health – disentangling value tensions. *J. Inf. Commun. Ethics Soc.* **17**, 229–245 (2019).

[16] Yeng, P. K., Fauzi, M. A. & Yang, B. A comprehensive assessment of human factors in cyber security compliance toward enhancing the security practice of healthcare staff in paperless hospitals. *Information* **13**, 335 (2022).

[17] Lanzing, M. Traveling technology and perverted logics: conceptualizing Palantir’s expansion into health as sphere transgression. *Inf. Commun. Soc.* **27**, 2617–2633 (2023).

[18] Fisher, B. & Tronto, J. Toward a feminist theory of caring. In *Circles of Care* (eds. Abel, E. K., Nelson, M. K.) 35–62 (State University of New York Press, 1990).

[19] Martin, A., Myers, N. & Viseu, A. The politics of care in technoscience. *Soc. Stud. Sci.* **45**, 625–641 (2015). 26630814 10.1177/0306312715602073

[20] Mol, A., Moser, I. & Pols, J. *Care in Practice* (Transcript, 2010).

[21] Rajamäki, J. & Hämäläinen, H. Ethics of cybersecurity and biomedical ethics: case SHAPES. *Inf. Secur.* **50**, 103–116 (2021).

[22] Mol, A. *The Logic of Care* (Routledge, 2008).

[23] Pols, J. & Moser, I. Cold technologies versus warm care? On affective and social relations with and through care technologies. *Alter* **3**, 159–178 (2009).

[24] Ghorayeb, A., Comber, R. & Gooberman-Hill, R. Older adults’ perspectives of smart home technology: are we developing the technology that older people want? *Int. J. Human Comput. Stud.* **147**. 10.1016/j.ijhcs.2020.102571 (2021).

[25] Renaud, K. Accessible cyber security: the next frontier? In *Proc. 7th international conference on information systems security and privacy*, (ed. Mori, P., Gabriele, L., Furnell, S.) 9–18. (SciTePress, 2020).

[26] Renaud, K. & Coles-Kemp, L. Accessible and inclusive cyber security: a nuanced and complex challenge. *SN Comput. Sci.* **3**, 346 (2022). 35755325 10.1007/s42979-022-01239-1 PMC9215151

[27] Mesman, J., Walsh, K., Kinsman, L., Ford, K. & Bywaters, D. Blending video-reflexive ethnography with solution-focused approach: a strengths-based approach to practice improvement in health care. *Int. J. Qual. Methods* **18**, 10.1177/1609406919875277 (2019).

[28] Hollnagel, E., Wears, R. L. & Braithwaite, J. From Safety-I to Safety-II: A White Paper. *Resilient Health Care Net*. 10.13140/RG.2.1.4051.5282 (2015).

[29] Oudshoorn, N. Diagnosis at a distance: the invisible work of patients and healthcare professionals in cardiac telemonitoring technology. *Sociol. Health Illn.* **30**, 272–288 (2008). 18290936 10.1111/j.1467-9566.2007.01032.x

[30] Trupia, D., Mathieu-Fritz, A. & Duong, T. The sociological perspective of users’ invisible work: a qualitative research framework for studying digital health innovations integration. *J. Med. Internet Res.* **23**, 1–13 (2021). 10.2196/25159 PMC8603174 34734832

[31] Koksch, L., Korn, M., Poller, A. & Wagenknecht, S. Caring for IT security: accountabilities, moralities, and oscillations in IT security practices. *Proc. ACM Hum. Comput. Interact.* **2**, 1–20 (2018). 92.

[32] FDA. *Applying human factors and usability engineering to medical devices* www.fda.gov/media/80481/download (2016).

[33] Verweij, L. et al. Improvement, implementation, and evaluation of the CMyLife digital care platform: participatory action research approach. *J. Med. Internet Res.* **25**, e45259 (2023). 37713242 10.2196/45259 PMC10541637

[34] De Croon, R., Klerkx, J. & Duval, E. Designing a useful and usable mobile EMR application through a participatory design methodology: a case study. In *2014 IEEE International Conference on Healthcare Informatics*, 176–185. (IEEE, 2014).

[35] Tang, K., Hirano, S., Cheng, K. & Hayes, G. Balancing caregiver and clinician needs in a mobile health informatics tool for preterm infants. In *Proc. 6th International Conference on Pervasive Computing Technologies for Healthcare*, 1–8 (IEEE, 2012).

[36] Kjörk, E., Sunnerhagen, K., Lundgren-Nilsson Å, Andersson, A. & Carlsson, G. Development of a digital tool for people with a long-term condition using stroke as a case example: participatory design approach. *JMIR Hum. Factors* **9**, e35478 (2022). 35657650 10.2196/35478 PMC9206198

[37] Brückner, S., Brightwell, C. & Gilbert, S. FDA launches health care at home initiative to drive equity in digital medical care. *npj Digital Med*. **7**. 10.1038/s41746-024-01198-2 (2024). 10.1038/s41746-024-01198-2 PMC11339355 39169161

[38] Slesinger, I., Coles-Kemp, L., Panteli, N. & Hansen, R. R. Designing through the stack: the case for a participatory digital security by design. In *Proc. 2022 New Security Paradigms Workshop*, 45–59. (ACM, 2022).

[39] Nhinda, G. T. & Shava, F. B. Towards the use of participatory methods in cybersecurity research in rural Africa: a grassroots approach. In *International Multidisciplinary Information Technology and Engineering Conference*, 1–7, (IEEE, 2021).

[40] Heckle, R. R. Security dilemma: healthcare clinicians at work. *IEEE Secur. Privacy* **9**, 14–19. (2011).

[41] Hollnagel, E., Woods, D. D. & Leveson, N. G. Resilience engineering: concepts and precepts (Ashgate, 2006).

[42] Goodair, B. & Reeves, A. The effect of health-care privatisation on the quality of care. *Lancet Public Health* **9**, e199–e206 (2024). 38429019 10.1016/S2468-2667(24)00003-3

[43] Roderick, P. & Pollock, A. M. Dismantling the National Health Service in England. *Int. J. Health Serv.* **52**, 470–479 (2022). 35876348 10.1177/00207314221114540 PMC9449439

[44] Hacker, J. S. Dismantling the Health Care State? Political institutions, public policies and the comparative politics of health reform. *Br. J. Political Sci.* **34**, 693–724 (2004).

